# Effect of Salinity and Plant Growth Promoters on Secondary Metabolism and Growth of Milk Thistle Ecotypes

**DOI:** 10.3390/life12101530

**Published:** 2022-09-30

**Authors:** Noreen Zahra, Abdul Wahid, Muhammad Bilal Hafeez, Irfana Lalarukh, Aaliya Batool, Muhammad Uzair, Mohamed A. El-Sheikh, Saleh Alansi, Prashant Kaushik

**Affiliations:** 1 Department of Botany, University of Agriculture, Faisalabad 38040, Pakistan; 2 Department of Botany, Government College for Women University, Faisalabad 38040, Pakistan; 3 Department of Agronomy, University of Agriculture, Faisalabad 38040, Pakistan; 4 Department of Plant Breeding and Genetics, University of Agriculture, Faisalabad 38040, Pakistan; 5 Botany and Microbiology Department, King Saud University, Riyadh 11451, Saudi Arabia; 6 Instituto de Conservación y Mejora de la Agrodiversidad Valenciana, Universitat Politècnica de València, 46022 Valencia, Spain

**Keywords:** milk thistle, secondary metabolites, ecotypes, salinity, growth attributes

## Abstract

**Simple Summary:**

The present study shed light on the effect of salinity on the plant growth and secondary metabolites of medicinally important milk thistle plant ecotypes. At the same time, we also studied the effect of external supplementation with ascorbic acid, thiourea, and moringa leaf extract on improving growth-related attributes and secondary metabolites under salinity stress. Various parameters were studied related to stress alleviation. Ascorbic acid, followed by moringa leaf extract, was the most effective in improving growth under salt stress conditions. The present study demonstrated that milk thistle could withstand moderate doses of salt stress, while externally supplemented media improved all the growth parameters by increasing the accumulation of secondary metabolites.

**Abstract:**

Milk thistle (*Silybum marianum* (L.)) is a wild medicinal herbal plant that is widely used in folk medicine due to its high content of secondary metabolites (SMs) and silymarin; however, the data regarding the response of milk thistle to salinity are still scarce and scanty. The present study evaluated the effect of salinity on a geographically diverse population of milk thistle and on the role of medium supplementation (MS) with ascorbic acid, thiourea, and moringa leaf extract in improving the SMs and growth-related attributes under salinity stress (SS). For germination, a 120 mM level of salinity was applied in the soil during the seedling stage. After salinity development, predetermined levels of the following compounds were used for MS: thiourea (250 µM), moringa leaf extract (3%), and ascorbic acid (500 µM). The data regarding growth attributes showed that SS impaired plant growth and development and increased SM production, including alkaloids, anthocyanin, and saponins. Moreover, ascorbic acid, followed by moringa leaf extract, was the most effective in improving growth by virtue of increased SMs, especially under salt stress conditions. The present study demonstrated that milk thistle could withstand moderate doses of SS, while MS improved all the growth parameters by increasing the accumulation of SMs.

## 1. Introduction

The cultivation of milk thistle has been increasing all over the world due to its uses in pharmaceutical industries [1]. The seeds of milk thistle contain bioactive compounds such as silychristin, silydianin, isosilybin, silybin, quercetin, apigenin, naringin, dihydrokaempferol, taxifolin, chrysoeriol, flavonolignans, eriodyctiol, and kaempferol [2,3,4]. It is used in pharmacologically relevant actions against different diseases, such as mushroom poisoning, liver injury due to drugs toxicities, and viral hepatitis, as reviewed by [5]. However, little attention has been paid to evaluating the effect of abiotic stresses on its morphology, growth, physio-chemical mechanisms, yield potential, and medium-supplementation-induced accumulation.

Globally, climate fluctuations are a foremost hazard to global food security for >7.8 billion people as of 2020 [6] and increase salinization [7,8], waterlogging [9], drought [9], and extreme temperatures [10,11]. Around the world, salinity stress (SS) affects over 20% of irrigated agricultural land, which is why it is considered as one of the key challenges for agricultural researchers [12,13,14,15,16]. Salinity stress causes osmotic and ionic stress, results in ionic homeostasis, which decreases growth and yield, and causes the premature senescence of leaves [17,18]. Salinity was found to reduce shoot and root length, and the relative growth rate of shoots and roots in wheat [19]. Similarly, under SS, leaf area, root length, fresh root weight, yield, and total soluble solids were found to be decreased in cherry tomato [20]. In pea, plant height, leaf area, plant fresh and dry weight, and relative water content were found to be decreased under SS [21]. Under SS, dry weight, shoot and root length, shoot and root numbers, leaf area, and relative water content in pomegranate were found to be negatively affected [22]. Under SS, the relative water content and secondary metabolites (total phenolic and total flavonoid contents) were decreased in sesame [23]. However, a maintained sugar production and the production of secondary metabolites could provide defense to ensure a more successful acclimation to SS [24,25,26].

Medium supplementation (MS) for plant growth was reported to promote the confronting of salinity stress and the overcoming of yield losses in all crops [27,28,29,30,31]. Medium supplementation with 24-brassinosteroid increased the canopy diameter, the length of branches, the diameter of branches, the total number of branches per tree, the number of branches producing fruit per tree, the total number of fruit setting per tree, and the total number of ripe fruit load per tree as compared with the control [32]. Medium supplementation with paclobutrazol increased grain yield, grain weight, and main panicle length in quinoa under SS [33]. Medium supplementation with uniconazole significantly increased plant height, stem, the length of spikes, and top dry weight in barley under SS [34]. Ascorbic acid is one of the plant growth promoters known as vitamin C (water-soluble) that deploys many physio-chemical modulations to provide tolerance against salinity [35]. Ascorbic acid treatment under SS increased shoot and root length and their fresh and dry weight, as well as nutrient elements, while it decreased lipid peroxidation [36]. Ascorbic acid application increased shoot height, leaf number, and tuberous root diameter under SS [37]. Thiourea is a plant growth promoter that scavenges ROS by modulating numerous essential functions under a plethora of abiotic stresses [38]. Exogenous treatment with thiourea enhanced shoot and root length, shoot and root fresh and dry weight, chlorophyll content, potassium, and zinc under SS [39]. Plant bio-stimulants such as moringa leaf extract trigger growth and increase crops’ economic yield because they are enriched with amino acids, growth hormones, antioxidants, vitamins, and mineral nutrients [40,41,42]. Moringa leaf extract application ameliorated SS by improving root and shoot fresh and dry weight, root and shoot length, potassium, calcium, and phosphorus [43]. The purpose of the current trial was to evaluate the efficacy of different plant growth promoters in modulating SMs and growth indicators in milk thistle ecotypes under saline stress conditions.

## 2. Materials and Methods

### 2.1. Experimental Details

A two-year pot trail was performed at “Botanical Garden, University of Agriculture, Faisalabad” to investigate the tolerance potential of different milk thistle ecotypes under SS. Different plant growth promoters were used to ameliorate the adversities of salt stress and for regulating silymarin biosynthesis. Plants of three ecotypes (Faisalabad, Gujranwala, and Quetta) were taken from varied geographic regions and sown in Faisalabad in an ecotype agronomic environment. The achenes of the F1 generation obtained from three ecotypes were sown on 17th November (2017–2018) under control conditions. The salinity level (120 mM) [44] was applied on 11th December, and different plant growth promoters were applied via MS. Plant growth promoters, such as thiourea (250 µM) [45], moringa leaf extract (3%) [46], and ascorbic acid (500 µM) [47], were applied at predetermined levels after inducing SS during the seedling stage through irrigation (single time point). Flowering started on 10th, 20th, and 25th February in the Faisalabad, Gujranwala, and Quetta ecotypes, respectively, in 2017, while in 2018, the flowering dates were 12th and 20th January for the Faisalabad and Gujranwala ecotypes, respectively, and 30th January for the Quetta ecotype. After harvest, all the morpho-physiological analyses were performed using standard protocols.

### 2.2. Growth and Yield Indicators

The careful harvesting of plants was performed on 11th April in both experimental years, and the samples were thoroughly rinsed with tap water. The shoot and root length was measured with a foot scale. After that, the plants shoots were detached from the roots, and the fresh weight (g) of shoots and roots were noted using an electric weight balance. The other indicators, such as the number of roots plant^−1^, the number of leaves plant^−1^, the leaf area (mm^2^), the root diameter (mm^2^), and the number of spines leaf^−1^, were also evaluated.

### 2.3. Determination of Secondary Metabolites

After sampling, fresh plants were instantly put in an ice bath and preserved at −40 °C in a laboratory for performing various biochemical analyses. Half of the cut shoots were moved to paper envelopes and dried for one week at 70 °C (Memert, Schwabach, Germany).

### 2.4. Anthocyanin Determination

The anthocyanin contents were estimated by following STRACK and WRAY [48]. For this, fresh plant samples (0.1 g) were homogenized in acidified methanol (2.5 mL). After that, samples were heated for 1 h (50 °C) and filtered. The optical density was noted at 535 nm.

### 2.5. Total Alkaloids Determination

The method by Singh and Sahu [49] was used to measure the total alkaloids contents. Plant material (0.1 g) was homogenized in 1 mL of methanol and then diluted with distilled water. For 1 mL of running sample, 0.5 mL of acetic acid and 0.01 M sodium meta periodate were added for each sample. The mixture was boiled, and after that, 0.01 M 3-methyl-2-benzothiazol solution was added to each test tube. The samples were cooled for 20 min in a water bath, and absorbance was recorded at 470 nm.

### 2.6. Saponin Estimation

For measuring the saponin contents, 0.1 g plant samples were homogenized until they were converted into powder form and then soaked in DH_2_O: ethanol in a one-to-one ratio. For 0.5 mL of extract, 5 mL of H_2_SO_4_ (72%) and 0.5 mL vanillin (10%) were added, while the sample mixtures were put on ice. The samples were moved to a water bath at 60 °C for 10 min. Absorbance was noted at 535 nm.

### 2.7. Statistical Analysis

The design of the trial was a Completely Randomized Factorial Design (CRD) with three replications. The main and interactive effects among SS, ecotypes, and MS under non-saline conditions were assessed using numerous response variables with the analysis of variance (ANOVA) technique at the 5% probability level. The ANOVA was performed using statistical software “Statistax8.1”. The trial data were also dealt with using a principal component analysis (PCA) using R-Stat to assess the existing relationships with original variables.

## 3. Results

### 3.1. Plant Length

**Figure 1:**
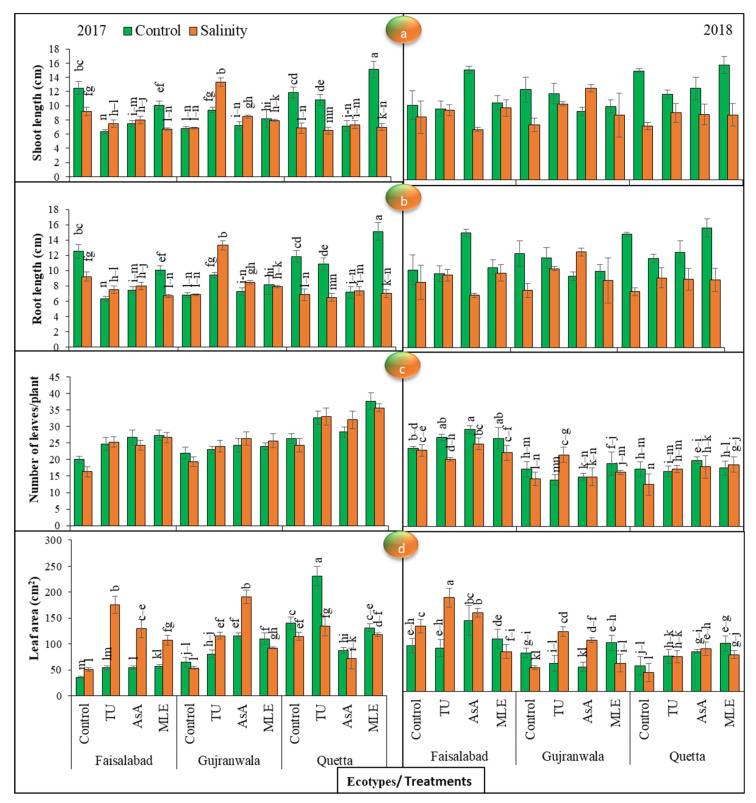
(**a**) Effects of MS with different plant growth promoters on shoot length, (**b**) root length, (**c**) number of leaves/plant, and (**d**) leaf area in milk thistle in 2017 and 2018. Different letters represent significant differences at the *p* < 0.05.

Significant (*p* < 0.01) differences were noted among all the factors for plant height (root and shoot length) in 2017. Under control conditions, the maximum shoot length were observed in the control, thiourea and moringa leaf extract treatment in the Faisalabad, Gujranwala, and Quetta ecotypes, respectively. Moreover, data displayed that under SS, the trends of the maximum increment in this parameter were found for the control, thiourea, and ascorbic acid treatments in the Faisalabad, Gujranwala, and Quetta ecotypes, respectively. Overall, Quetta performed well under control conditions; however, Gujranwala displayed the maximum shoot height under SS (Figure 1a). In 2018, MS with ascorbic acid, followed by moringa leaf extract, showed profound results for shoot length under non-saline conditions in the Faisalabad ecotype, while the effects of thiourea and moringa leaf extract were the most pronounced in the Gujranwala and Quetta ecotypes, respectively. Under SS, the effect of moringa leaf extract was the significantly increased the shoot length in the Faisalabad ecotype, while ascorbic acid was more effective in the Gujranwala and Quetta ecotypes than other plant growth promoters in 2018.

Considering the root length of control plants, the trends of the maximum root length following control, thiourea, and moringa leaf extract treatments were observed in the Faisalabad, Gujranwala, and Quetta ecotypes, respectively, in 2017. Moreover, while under SS conditions, MS with ascorbic acid performed better in the Faisalabad and Quetta ecotypes, and the effectiveness of thiourea was at its peak in Gujranwala. On the other hand, in 2018, an increasing trend for root length was observed in the order Quetta > Faisalabad > Gujranwala ecotypes. Under normal conditions, ascorbic acid showed the maximum increment in Faisalabad, while thiourea and moringa leaf extract showed the maximum increment in the Gujranwala and Quetta ecotypes, respectively. Under SS, moringa leaf extract showed the maximum increment in Faisalabad, while ascorbic acid and thiourea showed the maximum increments in the Gujranwala and Quetta ecotypes, respectively. Overall, Quetta performed well under control conditions; however, Gujranwala displayed the maximum shoot height under SS (Figure 1b).

### 3.2. Number of Leaves

Significant (*p* < 0.01) differences were recorded among all the factors for the number of leaves in 2018. In 2017, the trend of the maximum number of leaves was observed when moringa leaf extract was applied in all ecotypes under control conditions, while under SS conditions, MS with moringa leaf extract performed better in the Faisalabad and Quetta ecotypes, and the effectiveness of ascorbic acid was at its peak in Gujranwala. In 2018, the trends of the maximum number of leaves were observed following treatment with ascorbic acid in the Faisalabad and Quetta ecotypes, while moringa leaf extract treatment performed better in the Gujranwala ecotype under control conditions; on the other hand, under SS, the effect of ascorbic acid on the Faisalabad ecotype and the effects of thiourea and moringa leaf extract on the Gujranwala and Quetta ecotypes were more pronounced than those of MS with other plant growth promoters (Figure 1c).

### 3.3. Leaf Area

Significant (*p* < 0.01) differences were noted among all the factors for leaf area in both years. Under control conditions, the trends of the maximum leaf area were observed following treatments with moringa leaf extract and thiourea in Faisalabad, and in the Gujranwala and Quetta ecotypes, respectively, regardless of SS treatment in 2017, while under SS, the trends of leaf-area increment were observed following thiourea treatment in the Faisalabad and Gujranwala ecotypes and moringa leaf extract treatment in the Quetta ecotype. Moreover, ecotypic variation revealed the greatest leaf area in the Quetta ecotype. Furthermore, according to data recorded in 2018, the trend of the increment in this trait in the Faisalabad ecotype was observed with ascorbic acid, while in the Gujranwala and Quetta ecotypes, it was observed with moringa leaf extract. Under SS, this increasing trend was observed following thiourea treatment in the Faisalabad and Gujranwala ecotypes and following ascorbic acid treatment in the Quetta ecotype. Moreover, ecotypic variation revealed the greatest leaf area in the Quetta ecotype (Figure 1d).

### 3.4. Fresh Weight

**Figure 2:**
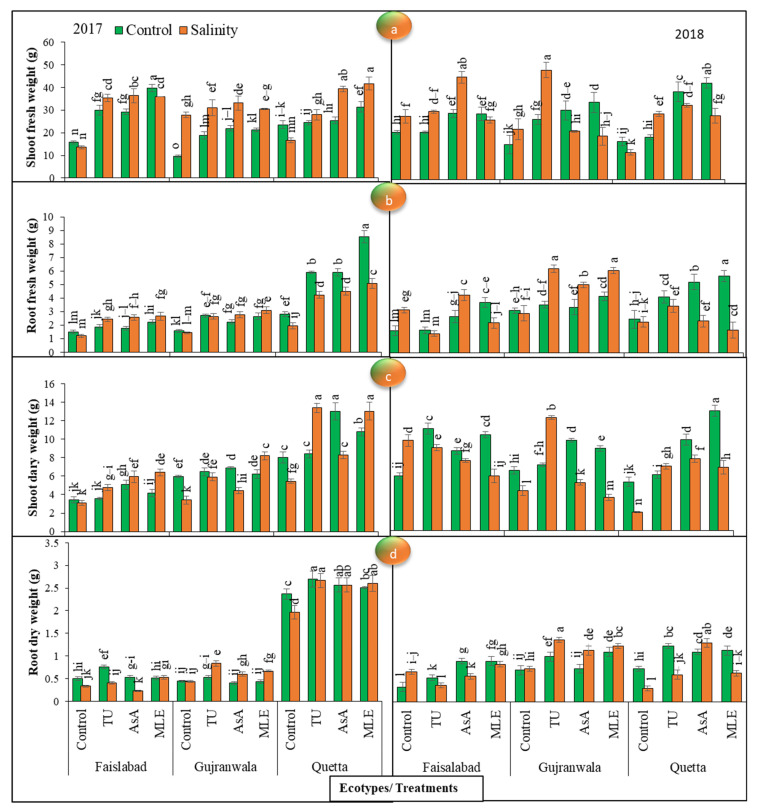
(**a**) Effects of MS with different plant growth promoters on shoot fresh weight, (**b**) root fresh weight, (**c**) shoot dry weight, and (**d**) root dry weight in milk thistle in 2017 and 2018. Different letters represent significant differences at the *p* < 0.05.

Plant fresh root and shoot weight displayed significant (*p* < 0.01) differences among all the factors in both trial years. Under non-saline conditions, the maximum increment in the Faisalabad and Quetta ecotypes was obtained by applying moringa leaf extract treatment; however, in Gujranwala, it was obtained with ascorbic acid treatment. Moreover, under SS, the maximum change in this attribute was obtained via MS with ascorbic acid in the Faisalabad and Gujranwala ecotypes, while in the Quetta ecotype, it was noted with moringa leaf extract treatment in 2017. MS with moringa leaf extract was the most effective in improving shoot fresh weight in all ecotypes under normal circumstances, while under SS, the effect of ascorbic acid treatment was the most pronounced in the Faisalabad and Quetta ecotypes, and in Gujranwala, thiourea-supplemented plants had the greatest fresh weight in 2018 (Figure 2a).

In 2017, the Faisalabad, Gujranwala, and Quetta ecotypes treated with moringa leaf extract had the greatest root fresh weight under control and SS conditions. Overall, under SS and normal conditions, the Quetta ecotype showed the maximum root fresh weight. In 2018, the maximum increment in this trait was obtained via MS with moringa leaf extract in all ecotypes, while under SS conditions, it was noted with ascorbic acid MS in Faisalabad, moringa leaf extract treatment = thiourea treatment in Gujranwala, and moringa leaf extract treatment in the Quetta ecotype (Figure 2b).

### 3.5. Dry Weight

Statistical data for plant dry weight (of shoots and roots) revealed significant (*p* < 0.01) differences among all the factors in both years. The data further revealed that ascorbic acid MS was effective in enhancing shoot dry weight in the first year of the experiment under normal conditions, while under SS conditions, the effects of moringa leaf extract MS were the most evident in all ecotypes. Additionally, the maximum shoot dry weight was verified in Quetta, followed by Gujranwala and Faisalabad, in 2017, while in 2018, under normal conditions, treatments with thiourea, ascorbic acid, and moringa leaf extract were more effective in the Faisalabad, Gujranwala, and Quetta ecotypes, respectively, than other treatments. Moreover, under SS conditions, the greatest shoot dry weight was observed with thiourea application in the Faisalabad and Gujranwala ecotypes, while ascorbic acid was effective in the Quetta ecotype. Furthermore, the greatest dry weight was noted in the Quetta ecotype regardless of the SS conditions in 2017–2018 (Figure 2c).

Data recorded for root dry weight depicted that thiourea MS showed the most pronounced results for dry weight under SS and control conditions in 2017. In 2018, the maximum increment obtained via MS with moringa leaf extract was observed in the Faisalabad and Gujranwala ecotypes, while thiourea treatment caused the maximum increment in the Quetta ecotype under control conditions. On the other hand, under SS, it was found that moringa leaf extract treatment in Faisalabad, thiourea treatment in Gujranwala, and ascorbic acid treatment in the Quetta ecotype showed the maximum root dry weight (Figure 2d). In conclusion, the data revealed that MS with plant growth promoters was quite efficient in accumulating the root dry weight in milk in all ecotypes under control and saline conditions, and the greatest dry weight was confirmed in the Quetta ecotype.

### 3.6. Number of Roots

**Figure 3:**
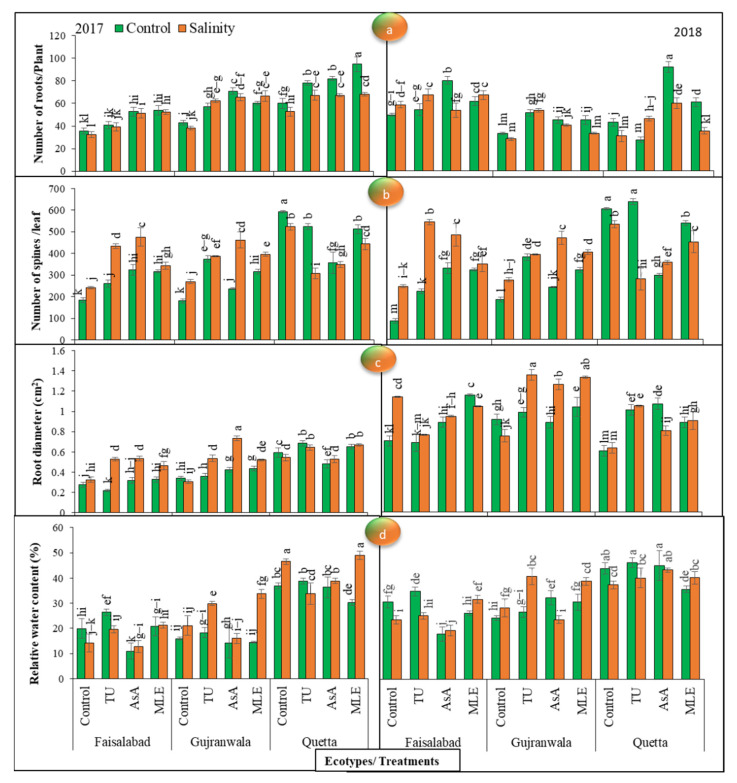
(**a**) Effects of MS with different plant growth promoters on number of roots/plant, (**b**) number of spines/leaf, (**c**) root diameter, and (**d**) relative water content in milk thistle in 2017 and 2018. Different letters represent significant differences at the *p* < 0.05.

The statistical data for the number of roots revealed significant (*p* < 0.01) differences among all the factors in both years. In 2017, an increasing trend in the Faisalabad and Quetta ecotypes was noted following moringa leaf extract treatment, while in the Gujranwala ecotype, this was observed following ascorbic acid treatment under control conditions. On the other hand, under saline conditions, the maximum number of roots was observed in all ecotypes via MS with moringa leaf extract. In 2018, an increasing trend under control conditions was observed following MS with ascorbic acid in the Faisalabad ecotype, while in the Gujranwala and Quetta ecotypes, it was observed following thiourea treatment. On the other hand, under SS, this increasing trend was observed following moringa leaf extract, thiourea, and ascorbic acid treatments in the Faisalabad, Gujranwala, and Quetta ecotypes, respectively (Figure 3a).

### 3.7. Number of Spines

The statistical data obtained for the number of spines displayed significant (*p* < 0.01) differences among all these factors and their interaction in both years. Under non-saline conditions, in 2017, the maximum increment in the number of spines was obtained via ascorbic acid, thiourea, and control treatments in the Faisalabad, Gujranwala, and Quetta ecotypes, respectively, while under SS conditions, the maximum increment in this parameter was obtained via MS with ascorbic acid in the Faisalabad and Gujranwala ecotypes and following control treatment in the Quetta ecotype. Furthermore, a higher number of spines was present on Quetta leaves than on the leaves of other ecotypes. In 2018, the maximum number of spines was observed following MS with ascorbic acid, which was the most effective in the Faisalabad ecotype, while thiourea was the most effective in the Gujranwala and Quetta ecotypes under normal circumstances; on the other hand, under SS, the effects of thiourea, ascorbic acid, and control treatments were the most efficient in the Faisalabad, Gujranwala, and Quetta ecotypes, respectively, and thiourea-supplemented plants had the highest number of spines in 2018. Interestingly, SS plants showed a greater number of spines than control plants of the Faisalabad and Gujranwala ecotypes, while an antagonistic effect was observed in the Quetta ecotype (Figure 3b).

### 3.8. Root Diameter

The results obtained for root diameter showed substantial (*p* < 0.01) variations among all the factors in both years. Under control conditions, the maximum change in 2017 was obtained via MS with moringa leaf extract, ascorbic acid, and thiourea in the Faisalabad, Gujranwala and Quetta ecotypes, respectively, while under SS, the change in this trait was observed following ascorbic acid treatment in all ecotypes. In 2018, the maximum increase in root diameter in non-stressed plants was obtained via MS with moringa leaf extract in the Faisalabad and Gujranwala ecotypes and via ascorbic acid treatment in the Quetta ecotype. However, under SS conditions, control treatment was effective in the Faisalabad ecotype, while thiourea treatment was effective in Gujranwala and Quetta. Furthermore, a greater root diameter was recorded in 2018 than in the previous year. Additionally, the greatest root diameter was measured in Gujranwala in 2017 and 2018 (Figure 3c).

### 3.9. Relative Water Content

The results found for the RWC exposed significant (*p* < 0.01) differences among all the factors in both years. Under normal conditions, in 2017, the increasing trend was at its maximum following MS with thiourea in all ecotypes as compared with other plant growth promoters. Meanwhile, under SS conditions, moringa leaf extract-supplied plants showed the maximum water content in all ecotypes. Considering the differences in the ecotypes revealed that the Quetta ecotype was efficient in accumulating higher relative water contents (Figure 3d).

The data noted in 2018 exhibited that the increasing trend in the Faisalabad and Quetta ecotypes was at its highest following MS with thiourea, while in Gujranwala, the application of ascorbic acid was the most effective under control conditions. However, under SS conditions, the maximum RWC was observed following MS with moringa leaf extract, thiourea, and ascorbic acid in the Faisalabad, Gujranwala, and Quetta ecotypes, respectively. The increasing trend with respect to the ecotypes was observed as: Quetta > Gujranwala > Faisalabad. Overall, the RWC increased with MS in the different ecotypes, while SS reduced the water content in the two trial studies.

### 3.10. Saponin Content

**Figure 4:**
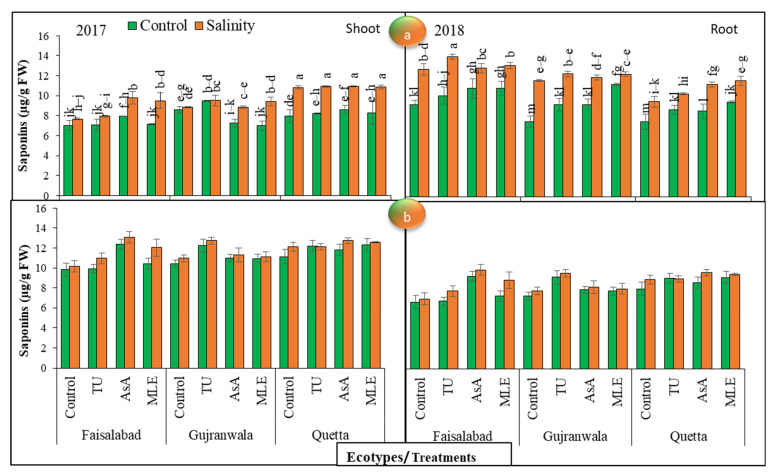
(**a**) Effects of MS with different plant growth promoters on shoot saponin contents and (**b**) root saponin contents in milk thistle in 2017 and 2018. Different letters represent significant differences at the *p* < 0.05.

The graphical data of saponin contents in both shoots and roots showed significant (*p* < 0.01) differences among all the factors in 2017. Under non-saline conditions, in 2017, the increasing trend in the Faisalabad and Quetta ecotypes with respect to shoot saponin was at its maximum with the soil addition of ascorbic acid, while in Gujranwala, this increase was at its maximum with the addition of thiourea under SS and control conditions. In 2018, ascorbic acid gave distinctive results in comparison with other plant growth promoters in the Faisalabad and Quetta ecotypes, while MS with thiourea was effective for increasing this trait at the maximum level in the Gujranwala ecotype under control and stress conditions (Figure 4a). The ecotypic differences showed the highest shoot saponin content in the Faisalabad ecotype in 2017, while in 2018, the maximum content was found in the Quetta ecotype regardless of SS. Regarding the root saponin content in control plants in 2017, ascorbic acid MS showed the highest value in Faisalabad, while moringa leaf extract MS showed distinctive results in the Gujranwala and Quetta ecotypes as compared with other plant growth promoters under control conditions. On the other hand, under SS, thiourea MS was the most effective in increasing the root saponin content in the Faisalabad and Gujranwala ecotypes, but in Quetta, moringa leaf extract was effective. In 2018, under control conditions, ascorbic acid gave distinctive results in comparison with other plant growth promoters in the Faisalabad and Quetta ecotypes, while thiourea MS was effective in increasing this trait at the maximum level in the Gujranwala ecotype. On the other hand, under SS, in 2018, under control conditions, ascorbic acid gave distinctive results in comparison with other plant growth promoters in the Faisalabad and Quetta ecotypes, while thiourea MS was effective in improving this trait at the maximum level in the Gujranwala ecotype. The data relating to this trait exposed that the lowest saponin content was recorded in Quetta, while the highest one was noted in Faisalabad, regardless of treatment differences (Figure 4b). Furthermore, SS maximally increased the saponin content with MS.

### 3.11. Anthocyanin Content

**Figure 5:**
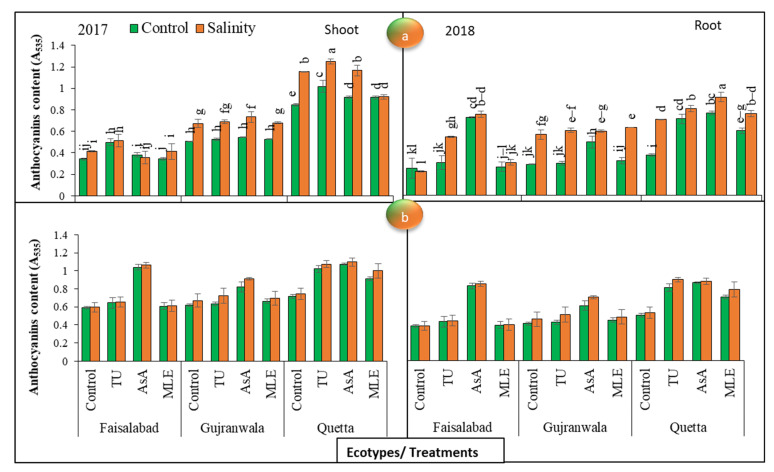
(**a**) Effects of MS with different plant growth promoters on shoot anthocyanin contents and (**b**) root anthocyanin contents in milk thistle in 2017 and 2018. Different letters represent significant differences at the *p* < 0.05.

The results obtained for anthocyanin contents showed significant (*p* < 0.01) differences among all the factors in 2017. Regarding the shoot anthocyanin content in 2017 under control conditions, the trends of the change in this trait were found to be maximum with thiourea MS in the Faisalabad and Quetta ecotypes, while the effect of plant growth promoters was lower in the Gujranwala ecotype. On the other hand, under SS, similar results were observed with respect to all ecotypes except for the Gujranwala ecotype, in which thiourea was the most effective in increasing anthocyanin. The data further revealed that in 2018, ascorbic acid MS showed the maximum increase in this trait under control and stress conditions. Moreover, the ecotypic variation showed an increasing trend for this trait as follows: Quetta > Gujranwala > Faisalabad in 2017. In 2018, it was: Quetta > Faisalabad > Gujranwala (Figure 5a).

The data noted for root anthocyanin contents exhibited that MS with ascorbic acid was the most effective in increasing the anthocyanin content in both years, regardless of ecotypic variations and SS. The trends of the change in this trait with respect to the ecotypes was found to be Quetta > Faisalabad > Gujranwala (Figure 5b).

In conclusion, the results exhibited that SS improved the anthocyanin content in both trial years, and MS application increased the anthocyanin content to a significant extent, regardless of SS and the differences in the ecotypes. Moreover, shoots and the 2017 year were superior in synthesizing more anthocyanin with respect to roots and the 2018 year, respectively.

### 3.12. Alkaloids Content

**Figure 6:**
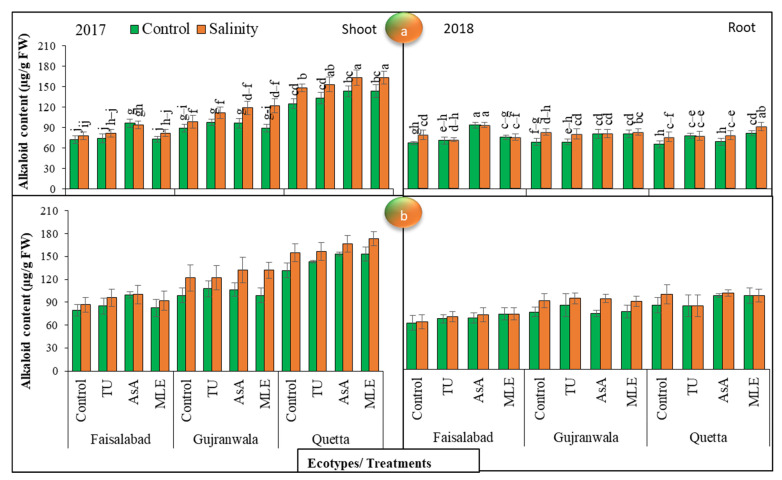
(**a**) Effects of MS with different plant growth promoters on shoot alkaloids contents and (**b**) root alkaloids contents in milk thistle in 2017 and 2018. Different letters represent significant differences at the *p* < 0.05.

The alkaloids contents demonstrated significant (*p* < 0.01) differences among all these factors, which were also highly significant in 2017, for both root and shoot parts. Under control conditions, regarding the shoot alkaloids content in 2017, ascorbic acid MS showed the highest value in the Faisalabad and Quetta ecotypes, while thiourea MS = ascorbic acid MS in terms of effectiveness in the Gujranwala ecotype. On the other hand, under SS, ascorbic acid showed the highest value in Faisalabad, and moringa leaf extract = ascorbic acid in the Quetta ecotype, while thiourea MS = ascorbic acid MS in terms of showing the maximum results in Gujranwala. The data further revealed that in 2018, the effect of ascorbic acid was the most pronounced on the Faisalabad and Quetta ecotypes, while thiourea had the greatest effect on increasing this trait in the Gujranwala ecotype under control conditions. On the other hand, under SS, ascorbic acid MS was effective in the Faisalabad and Gujranwala ecotypes, while in the Quetta ecotype, moringa leaf extract MS gave the maximum increase as compared with other plant growth promoters (Figure 6a).

The data noted for root alkaloids contents depicted that ascorbic acid MS had the greatest effect on the Faisalabad ecotype in improving this attribute, while in the Gujranwala and Quetta ecotypes, moringa leaf extract MS was the most effective under SS and control stress conditions in 2017. On the other hand, in 2018, the maximum root alkaloids content in the Faisalabad ecotype was obtained via MS with moringa leaf extract, in the Gujranwala ecotype via MS with thiourea, and in Quetta via MS with ascorbic acid under non-saline conditions, while under SS conditions, ascorbic acid MS was the most effective in increasing the alkaloids content in the Faisalabad and Quetta ecotypes, and in Gujranwala, thiourea was the most effective. In contrast, the root alkaloids content was the highest in Faisalabad, followed by Quetta and Gujranwala, in 2017, while in 2018, it was Quetta > Gujranwala > Faisalabad. The alkaloids content was higher in shoots than in roots (Figure 6b).

### 3.13. Principle Component Analysis

**Figure 7:**
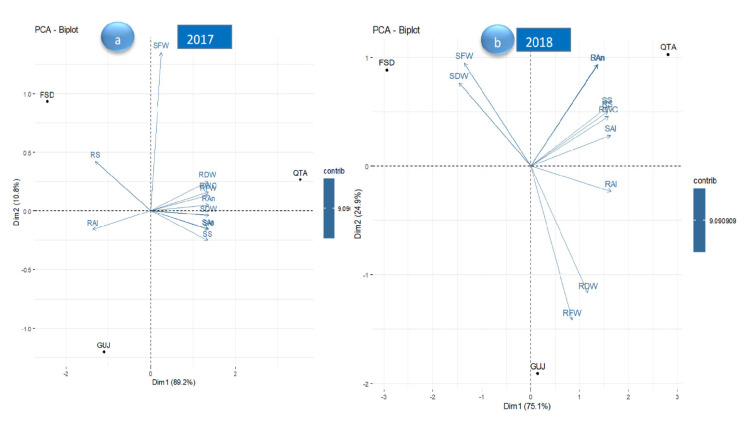
Principal component biplot of three ecotypes for all the traits under study in years (**a**) 2017 and (**b**) 2018. RFW = root fresh weight; SFW = shoot fresh weight; RDW = root dry weight; SDW = shoot dry weight; RAl = root alkaloids; SAl = shoot alkaloids; RAn = root anthocyanins; SAn = shoot anthocyanins; RS = root saponins; SS = shoot saponins; RWC = relative water content.

For the year 2017, both principal components 1 and 2 explained all the variation, i.e., 100% of the data. On average, the Quetta ecotype performed best for all the traits under study. It performed negatively for root saponins and root alkaloids but positively for all the other traits. However, the Faisalabad ecotype performed best for shoot fresh weight and root saponins but negatively for all the other traits. Similarly, the Gujranwala ecotype performed best for root alkaloids but negatively for all the other traits. In addition, the contribution of all parameters to the total variation is shown by the intensity of the vectors’ color (Figure 7a).

For the year 2018, both principal components 1 and 2 explained all the variation, i.e., 100% of the data. On average, the Quetta ecotype performed best for all the traits. It performed negatively for both fresh and dry weight (shoots + roots) but positively for all the other traits. However, the Faisalabad ecotype performed best for shoot weight (fresh + dry) but negatively for all the other traits. Similarly, the Gujranwala ecotype performed best for root weight (fresh + dry) but negatively for all the other traits. In addition, the contribution of all parameters to the total variation is shown by the intensity of the vectors’ color (Figure 7b).

## 4. Discussion

The enzymes concerning nitrogen and carbon metabolism are significantly altered under salinity stress, thereby resulting in a lower production of SMs and hampering plant growth [50]. By observing milk thistle performance, it was found that the shoot and root length in milk thistle planted in saline soil was shorter (Figure 1a,b) and that leaf color was slightly changed to yellowish-green as compared with control conditions. Overall, the data showed that salt stress reduced the vegetative growth of milk thistle, such as the number of leaves (Figure 1c), leaf area (Figure 1d), shoot and root fresh and dry weight (Figure 2), the number of roots (Figure 3a), and the RWC (Figure 3d). The present study’s thiourea results confirmed the studies of [51,52]; they also observed a significant reduction in plant length, the number of leaves, and root and shoot fresh weight. In another case [53], it was noted that plant height increased in *Alhagi pseudoalhagi* (a leguminous plant) at 5 dS/m, while it decreased at 10 and 20 dS/m. In another study of thiourea, [54] confirmed milk thistle growth reduction at 9 dS/m. However, in this study, increases in the number of spines and root diameter were seen, which confirmed the anticipatory role of salt stress (Figure 3b,c). According to [55], SS increased root area by up to 20% in *Brassica napus*, which indicated the spontaneous response of plants consisting in the uptake of more nutrients and water under stressful conditions. Contrarily, [56] observed a root-surface-area reduction in wheat under SS. Additionally, the application of plant growth promoters significantly increased all growth-related attributes regardless of salinity treatment, and an increasing trend was observed as follows: ascorbic acid > moringa leaf extract > thiourea. Our studies were inconsistent with the findings obtained by [57,58]; they also observed higher growth rate and RWC in maize with the application of the above-mentioned plant growth promoters. They suggested that ascorbic acid and thiourea improved growth-related attributes by prompting the photosynthetic capacity. Moringa leaf extract is also a rich source of ascorbate, which was found to promote a shielding effect against oxidative stress and to improve photosynthetic efficiency [59]. On the other hand, ecotypic variations showed the highest growth rate in Quetta, followed by Gujranwala and Faisalabad.

According to the observations in [60], SMs increased in safflower under saline stress conditions at 5 to 15 dS/m. In this study, a higher SM content in terms of anthocyanin was observed both under SS and in plants supplied with plant growth promoters than in unstressed plants (Figure 5). The authors of [61] reported that the anthocyanin content increased in response to salinity, while it decreased in salt-sensitive genotypes. According to [62], the anthocyanin content increased in wheat genotypes under salt stress conditions. In another study, [63] reported higher anthocyanin contents in tomato and cabbage under SS. In addition, higher total alkaloid and saponin (Figure 4 and Figure 6) contents were observed under salt stress conditions, and MS with plant growth promoters further increased their concentrations, regardless of ecotypic variations. The authors of [64] also deduced that the alkaloids content increased in *Chelidinium majus* L. in response to drought and SS. They presupposed that this increase might have been due to the increased enzymatic activities of stylopine synthase, which takes part in alkaloid biosynthesis. In contrast, [65] found a decreased content of alkaloids in *Catharanthus roseus* (L.) at 5 to 15 dS/m salinity. In another case, [66] observed higher alkaloid and saponin contents in soybean under SS. The effect of ascorbic acid, followed by moringa leaf extract and thiourea, was the most pronounced in enhancing the SM contents in all milk thistle ecotypes. Our findings are corroborated by the results of [67]; they also observed higher SM production in common bean with the application of ascorbic acid (200 or 400 mg L^−1^) under SS conditions, which may have been directly or indirectly linked with its antioxidative properties. Ecotypic variations showed higher SM contents in Quetta ecotypes than in other ecotypes, regardless of SS and MS with plant growth promoters. In addition, a higher alkaloids content was recorded in 2018 than in 2017, while saponin and anthocyanin contents were higher in 2017 under net house conditions. Therefore, using agronomic practices such as MS with plant growth promoters, especially ascorbic acid in Quetta ecotype, can enhance SS tolerance and could be used as a key for improving the SMs required for sustainable, low-input production in the SS environments of the world.

## 5. Conclusions

SS imprinted a negative effect on all growth-related attributes of milk thistle except for the number of spines and root diameter, which were surprisingly increased under SS treatment. The harmful effects of SS could be ameliorated via MS with plant growth promoters ascorbic acid, thiourea, and moringa leaf extract in milk thistle plants. Additionally, salt stress increased the synthesis of SMs, as confirmed by the increases in total alkaloids, saponin, and anthocyanin contents in SS-exposed plants, while their production was further enhanced via MS with plant growth promoters. Regarding ecotypic variations, the Quetta ecotype performed better not only under control but also SS conditions. Overall, a higher growth rate was observed in 2018 than in 2017. In a nutshell, an adequate supply of plant growth promoters played the anticipated role in improving the salinity tolerance of milk thistle plants by increasing the production of SMs.

## Data Availability

The data analyzed for this paper can be requested from the corresponding author.

## References

[1] Shah M., Jan H., Drouet S., Tungmunnithum D., Shirazi J.H., Hano C., Abbasi B.H. (2021). Chitosan Elicitation Impacts Flavonolignan Biosynthesis in *Silybum marianum* (L.) Gaertn Cell Suspension and Enhances Antioxidant and Anti-Inflammatory Activities of Cell Extracts. Molecules.

[2] Kim N.-C., Graf T.N., Sparacino C.M., Wani M.C., Wall M.E. (2003). Complete isolation and characterization of silybins and isosilybins from milk thistle (*Silybum marianum*). Org. Biomol. Chem..

[3] Denev P., Ognyanov M., Georgiev Y., Teneva D., Klisurova D., Yanakieva I.Z. (2020). Chemical composition and antioxidant activity of partially defatted milk thistle (*Silybum marianum* L.) seeds. Bulg. Chem. Commun..

[4] Choe U., Whent M., Luo Y., Yu L. (2020). Total phenolic content, free radical scavenging capacity, and anti-cancer activity of silymarin. J. Food Bioact..

[5] Abenavoli L., Izzo A.A., Milić N., Cicala C., Santini A., Capasso R. (2018). Milk thistle (*Silybum marianum*): A concise overview on its chemistry, pharmacological, and nutraceutical uses in liver diseases. Phytother. Res..

[6] Kogo B.K., Kumar L., Koech R. (2020). Climate change and variability in Kenya: A review of impacts on agriculture and food security. Environ. Develop. Sustain..

[7] Hafeez M.B., Raza A., Zahra N., Shaukat K., Akram M.Z., Iqbal S., Basra S.M.A. (2021). Gene regulation in halophytes in conferring salt tolerance. Handbook of Bioremediation.

[8] Kaya C., Ashraf M., Alyemeni M.N., Ahmad P. (2020). The role of endogenous nitric oxide in salicylic acid-induced up-regulation of ascorbate-glutathione cycle involved in salinity tolerance of pepper (*Capsicum annuum* L.) plants. Plant Physiol. Biochem..

[9] Zahra N., Hafeez M.B., Shaukat K., Wahid A., Hussain S., Naseer R., Raza A., Iqbal S., Farooq M. (2021). Hypoxia and Anoxia Stress: Plant responses and tolerance mechanisms. J. Agron. Crop Sci..

[10] Mubarik M.S., Khan S.H., Sajjad M., Raza A., Hafeez M.B., Yasmeen T., Rizwan M., Ali S., Arif M.S. (2021). A manipulative interplay between positive and negative regulators of phytohormones: A way forward for improving drought tolerance in plants. Physiol. Plant..

[11] Batool S., Khan S., Basra S.M., Hussain M., Saddiq M.S., Iqbal S., Irshad S., Hafeez M. (2019). Impact of natural and synthetic plant stimulants on Moringa seedlings grown under low-temperature conditions. Int. Lett. Nat. Sci..

[12] Akhter M.S., Noreen S., Mahmood S., Ashraf M., Alsahli A.A., Ahmad P. (2021). Influence of salinity stress on PSII in barley (*Hordeum vulgare* L.) genotypes, probed by chlorophyll-a fluorescence. J. King Saud Univ. Sci..

[13] Ahmad P., Venema K., Corpas F.J. (2022). Unravelling salt tolerance mechanisms in plants: From Lab to Field. Plant Physiol. Biochem..

[14] Ahammed G.J., Li Y., Li X., Han W.-Y., Chen S. (2018). Epigallocatechin-3-gallate alleviates salinity-retarded seed germination and oxidative stress in tomato. J. Plant Growth Regul..

[15] Shah T., Latif S., Saeed F., Ali I., Ullah S., Alsahli A.A., Jan S., Ahmad P. (2020). Seed priming with titanium dioxide nanoparticles enhances seed vigor, leaf water status, and antioxidant enzyme activities in maize (*Zea mays* L.) under salinity stress. J King Saud Uni. Sci..

[16] Ahanger M.A., Mir R.A., Alyemeni M.N., Ahmad P. (2020). Combined effects of brassinosteroid and kinetin mitigates salinity stress in tomato through the modulation of antioxidant and osmolyte metabolism. Plant Physiol. Biochem..

[17] Zahra N., Raza Z.A., Mahmood S. (2020). Effect of salinity stress on various growth and physiological attributes of two contrasting maize genotypes. Brazil. Arch. Biol. Technol..

[18] Arif Y., Singh P., Siddiqui H., Bajguz A., Hayat S. (2020). Salinity induced physiological and biochemical changes in plants: An omic approach towards salt stress tolerance. Plant Physiol. Biochem..

[19] Saddiq M.S., Iqbal S., Hafeez M.B., Ibrahim A.M., Raza A., Fatima E.M., Baloch H., Woodrow P., Ciarmiello L.F. (2021). Effect of salinity stress on physiological changes in winter and spring wheat. Agronomy.

[20] El-Beltagi H.S., Ahmad I., Basit A., El-Lateef A., Hany M., Yasir M., Tanveer Shah S., Ullah I., Elsayed Mohamed Mohamed M., Ali I. (2022). Effect of azospirillum and azotobacter species on the performance of cherry tomato under different salinity levels. Gesunde Pflanz..

[21] Ismail L.M., Soliman M.I., Abd El-Aziz M.H., Abdel-Aziz H.M. (2022). Impact of silica ions and nano silica on growth and productivity of pea plants under salinity stress. Plants.

[22] Liu C., Zhao X., Yan J., Yuan Z., Gu M. (2020). Effects of salt stress on growth, photosynthesis, and mineral nutrients of 18 pomegranate (*Punica granatum*) cultivars. Agronomy.

[23] Khademian R., Asghari B., Sedaghati B., Yaghoubian Y. (2019). Plant beneficial rhizospheric microorganisms (PBRMs) mitigate deleterious effects of salinity in sesame (*Sesamum indicum* L.): Physio-biochemical properties, fatty acids composition and secondary metabolites content. Ind. Crops Prod..

[24] MacNeill G.J., Mehrpouyan S., Minow M.A., Patterson J.A., Tetlow I.J., Emes M.J., Raines C. (2017). Starch as a source, starch as a sink: The bifunctional role of starch in carbon allocation. J. Exp. Bot..

[25] Hasanuzzaman M., Bhuyan M., Zulfiqar F., Raza A., Mohsin S.M., Mahmud J.A., Fujita M., Fotopoulos V. (2020). Reactive oxygen species and antioxidant defense in plants under abiotic stress: Revisiting the crucial role of a universal defense regulator. Antioxidants.

[26] Khaliq A., Zia-ul-Haq M., Ali F., Aslam F., Matloob A., Navab A., Hussain S. (2015). Salinity tolerance in wheat cultivars is related to enhanced activities of enzymatic antioxidants and reduced lipid peroxidation. CLEAN–Soil Air Water.

[27] Kamiab F. (2020). Exogenous melatonin mitigates the salinity damages and improves the growth of pistachio under salinity stress. J. Plant Nutr..

[28] Ishaq H., Nawaz M., Azeem M., Mehwish M., Naseem M.B.B. (2021). Ascorbic Acid (Asa) improves Salinity Tolerance in Wheat (*Triticum Aestivum* L.) by Modulating Growth and Physiological Attributes. J. Bioresour. Manag..

[29] Ahmadi F., Karimi K., Struik P. (2018). Effect of exogenous application of methyl jasmonate on physiological and biochemical characteristics of *Brassica napus* L. cv. Talaye under salinity stress. S. Afr. J. Bot..

[30] Alam P., Albalawi T.H., Altalayan F.H., Bakht M.A., Ahanger M.A., Raja V., Ashraf M., Ahmad P. (2019). 24-Epibrassinolide (EBR) confers tolerance against NaCl stress in soybean plants by up-regulating antioxidant system, ascorbate-glutathione cycle, and glyoxalase system. Biomolecules.

[31] Ahanger M.A., Aziz U., Alsahli A.A., Alyemeni M.N., Ahmad P. (2020). Influence of exogenous salicylic acid and nitric oxide on growth, photosynthesis, and ascorbate-glutathione cycle in salt stressed *Vigna angularis*. Biomolecules.

[32] Zheng Y., Xu B., Ren K., Zhang Y., Wu J. (2017). Impact of soil drench and foliar spray of 24-epibrassinolide on the growth, yield, and quality of field-grown *Moringa oleifera* in Southwest China. J. Plant Growth Regul..

[33] Waqas M., Yaning C., Iqbal H., Shareef M., ur Rehman H., Iqbal S., Mahmood S. (2019). Soil drenching of paclobutrazol: An efficient way to improve quinoa performance under salinity. Physiol. Plant..

[34] Hussein M., Bakheta M., Zaki S. (2014). Influence of uniconazole on growth characters, photosynthetic pigments, total carbohydrates and total soluble sugars of *Hordium vulgare* L. plants grown under salinity stress. Int. J. Sci. Res..

[35] Akram N.A., Shafiq F., Ashraf M. (2017). Ascorbic acid-a potential oxidant scavenger and its role in plant development and abiotic stress tolerance. Front. Plant Sci..

[36] Wang Y.-H., Zhang G., Chen Y., Gao J., Sun Y.-R., Sun M.-F., Chen J.-P. (2019). Exogenous application of gibberellic acid and ascorbic acid improved tolerance of okra seedlings to NaCl stress. Acta Physiol. Plant..

[37] de Sousa Basílio A.G., Vieira de Sousa L., da Silva T.I., de Moura J.G., de Melo Gonçalves A.C., de Melo Filho J.S., Leal Y.H., Jardelino Dias T. (2018). Radish (*Raphanus sativus* L.) morphophysiology under salinity stress and ascorbic acid treatments. Agron. Colomb..

[38] Waqas M.A., Kaya C., Riaz A., Farooq M., Nawaz I., Wilkes A., Li Y. (2019). Potential mechanisms of abiotic stress tolerance in crop plants induced by thiourea. Front. Plant Sci..

[39] Aziz U., Qadir I., Yasin G., Azhar M.F., Javed A., Akhtar A. (2021). Potential of priming in improving germination, seedling growth and nutrient status of Calotropis procera under salinity. Pak. J. Bot..

[40] Yaseen A., Takacsne Hajos M. (2020). Study on moringa tree (*Moringa oleifera* Lam.) leaf extract in organic vegetable production: A review. Res. Crops.

[41] Faisal M., Iqbal S., Basra S., Afzal I., Saddiq M., Bakhtavar M., Hafeez M., Rehman H., Basit A., Habib-ur-Rahman M. (2020). Moringa landraces of Pakistan are potential source of premium quality oil. S. Afr. J. Bot..

[42] Aslam M.F., Basra S.M., Hafeez M.B., Khan S., Irshad S., Iqbal S., Saqqid M.S., Akram M.Z. (2020). Inorganic fertilization improves quality and biomass of *Moringa oleifera* L.. Agrofor. Syst..

[43] Ahmed T., Abou Elezz A., Khalid M.F. (2021). Hydropriming with moringa leaf extract mitigates salt stress in Wheat seedlings. Agriculture.

[44] Yaghoubian I., Antar M., Ghassemi S., Modarres-Sanavy S.A.M., Smith D.L. (2022). The Effects of Hydro-Priming and Colonization with *Piriformospora indica* and *Azotobacter chroococcum* on Physio-Biochemical Traits, Flavonolignans and Fatty Acids Composition of Milk Thistle (*Silybum marianum*) under Saline Conditions. Plants.

[45] Perveen A., Wahid A., Mahmood S., Hussain I., Rasheed R. (2015). Possible mechanism of medium-supplemented thiourea in improving growth, gas exchange, and photosynthetic pigments in cadmium-stressed maize (*Zea mays*). Brazil. J. Bot..

[46] Rashid N., Basra S.M., Shahbaz M., Iqbal S., Hafeez M.B. (2018). Foliar applied moringa leaf extract induces terminal heat tolerance in quinoa. Int. J. Agric. Biol..

[47] Peñas E., Limón R.I., Martínez-Villaluenga C., Restani P., Pihlanto A., Frias J. (2015). Impact of elicitation on antioxidant and potential antihypertensive properties of lentil sprouts. Plant Foods Human Nutr..

[48] STRACK D., WRAY V. (1989). Anthocyanins. Methods in Plant Biochemistry.

[49] Singh D., Sahu A. (2006). Spectrophotometric determination of caffeine and theophylline in pure alkaloids and its application in pharmaceutical formulations. Anal. Biochem..

[50] Marino D., González E.M., Arrese-Igor C. (2006). Drought effects on carbon and nitrogen metabolism of pea nodules can be mimicked by paraquat: Evidence for the occurrence of two regulation pathways under oxidative stresses. J. Exp. Bot..

[51] Su Y., Guo A., Huang Y., Wang Y., Hua J. (2020). GhCIPK6a increases salt tolerance in transgenic upland cotton by involving in ROS scavenging and MAPK signaling pathways. BMC Plant Biol..

[52] Chartzoulakis K., Klapaki G. (2000). Response of two greenhouse pepper hybrids to NaCl salinity during different growth stages. Sci. Horti..

[53] Kurban H., Saneoka H., Nehira K., Adilla R., Premachandra G.S., Fujita K. (1999). Effect of salinity on growth, photosynthesis and mineral composition in leguminous plant *Alhagi pseudoalhagi* (Bieb.). Soil Sci. Plant Nutr..

[54] Ghavami N., Ramin A. (2008). Grain yield and active substances of milk thistle as affected by soil salinity. Comm. Soil Sci. Plant Anal..

[55] Arif M.R., Islam M.T., Robin A.H.K. (2019). Salinity stress alters root morphology and root hair traits in *Brassica napus*. Plants.

[56] Robin A.H.K., Matthew C., Uddin M.J., Bayazid K.N. (2016). Salinity-induced reduction in root surface area and changes in major root and shoot traits at the phytomer level in wheat. J. Exp. Bot..

[57] Sahu M., Solanki N., Dashora L. (1993). Effects of thiourea, thiamine and ascorbic acid on growth and yield of maize (*Zea mays* L.). J. Agron. Crop Sci..

[58] Waqas M.A., Khan I., Akhter M.J., Noor M.A., Ashraf U. (2017). Exogenous application of plant growth regulators (PGRs) induces chilling tolerance in short-duration hybrid maize. Environ. Sci. Pollut. Res..

[59] Khan A., Ashraf M. (2008). Exogenously applied ascorbic acid alleviates salt-induced oxidative stress in wheat. Environ. Exp. Bot..

[60] Gengmao Z., Yu H., Xing S., Shihui L., Quanmei S., Changhai W. (2015). Salinity stress increases secondary metabolites and enzyme activity in safflower. Ind. Crops Prod..

[61] Liang W., Ma X., Wan P., Liu L. (2018). Plant salt-tolerance mechanism: A review. Biochem. Biophys. Res. Comm..

[62] Mbarki S., Sytar O., Zivcak M., Abdelly C., Cerda A., Brestic M. (2018). Anthocyanins of Coloured wheat genotypes in specific response to SalStress. Molecules.

[63] Eryılmaz F. (2006). The relationships between salt stress and anthocyanin content in higher plants. Biotechnol. Biotechnol. Equip..

[64] Yahyazadeh M., Meinen R., Hänsch R., Abouzeid S., Selmar D. (2018). Impact of drought and salt stress on the biosynthesis of alkaloids in *Chelidonium majus* L.. Phytochemistry.

[65] Idrees M., Naeem M., Aftab T., Khan M.M.A. (2011). Salicylic acid mitigates salinity stress by improving antioxidant defence system and enhances vincristine and vinblastine alkaloids production in periwinkle [*Catharanthus roseus* (L.) G. Don]. Acta Physiol. Plant..

[66] Radhakrishnan R., Leelapriya T., Kumari B.D.R. (2012). Effects of pulsed magnetic field treatment of soybean seeds on calli growth, cell damage, and biochemical changes under salt stress. Bioelectromagnetics.

[67] Gaafar A.A., Ali S.I., El-Shawadfy M.A., Salama Z.A., Sekara A., Ulrichs C., Abdelhamid M.T. (2020). Ascorbic acid induces the increase of secondary metabolites, antioxidant activity, growth, and productivity of the common bean under water stress conditions. Plants.

